# Ionic Strength and Solution Composition Dictate the Adsorption of Cell-Penetrating Peptides onto Phosphatidylcholine Membranes

**DOI:** 10.1021/acs.langmuir.2c01435

**Published:** 2022-09-09

**Authors:** Man Thi Hong Nguyen, Denys Biriukov, Carmelo Tempra, Katarina Baxova, Hector Martinez-Seara, Hüseyin Evci, Vandana Singh, Radek Šachl, Martin Hof, Pavel Jungwirth, Matti Javanainen, Mario Vazdar

**Affiliations:** † Institute of Organic Chemistry and Biochemistry of the Czech Academy of Sciences, Flemingovo nám. 542/2, CZ-16000 Prague 6, Czech Republic; ‡ J. Heyrovský Institute of Physical Chemistry of the Czech Academy of Sciences, Dolejškova 2155/3, CZ-18223 Prague 8, Czech Republic; ¶ Department of Chemistry, Faculty of Science, University of South Bohemia in Ceske Budejovice, 370 05 Ceske Budejovice, Czech Republic; § Faculty of Mathematics and Physics at Charles University, 110 00 Prague, Czech Republic; ∥ Institute of Biotechnology, University of Helsinki, FI-00014 University of Helsinki, Finland; ⊥ Department of Mathematics, University of Chemistry and Technology, 166 28 Prague, Czech Republic

## Abstract

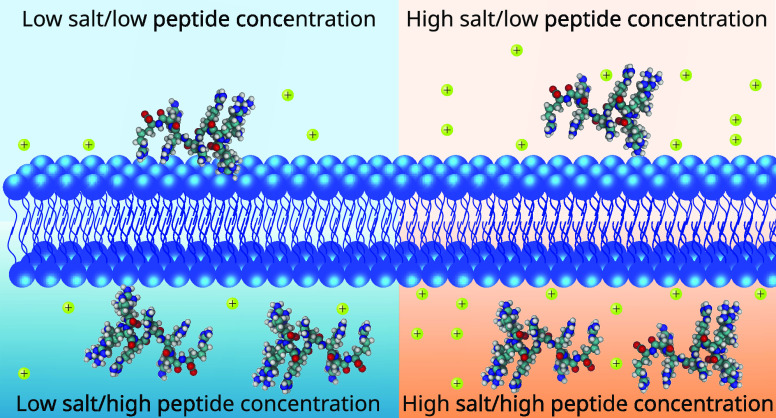

Adsorption of arginine-rich positively charged peptides onto neutral zwitterionic phosphocholine (PC) bilayers is a key step in the translocation of those potent cell-penetrating peptides into the cell interior. In the past, we have shown both theoretically and experimentally that polyarginines adsorb to the neutral PC-supported lipid bilayers in contrast to polylysines. However, comparing our results with previous studies showed that the results often do not match even at the qualitative level. The adsorption of arginine-rich peptides onto 1-palmitoyl-2-oleoyl-*sn*-glycero-3-phosphocholine (POPC) may qualitatively depend on the actual experimental conditions where binding experiments have been performed. In this work, we systematically studied the adsorption of R_9_ and K_9_ peptides onto the POPC bilayer, aided by molecular dynamics (MD) simulations and fluorescence cross-correlation spectroscopy (FCCS) experiments. Using MD simulations, we tested a series of increasing peptide concentrations, in parallel with increasing Na^+^ and Ca^2+^ salt concentrations, showing that the apparent strength of adsorption of R_9_ decreases upon the increase of peptide or salt concentration in the system. The key result from the simulations is that the salt concentrations used experimentally can alter the picture of peptide adsorption qualitatively. Using FCCS experiments with fluorescently labeled R_9_ and K_9_, we first demonstrated that the binding of R_9_ to POPC is tighter by almost 2 orders of magnitude compared to that of K_9_. Finally, upon the addition of an excess of either Na^+^ or Ca^2+^ ions with R_9_, the total fluorescence correlation signal is lost, which implies the unbinding of R_9_ from the PC bilayer, in agreement with our predictions from MD simulations.

## Introduction

The inefficient transfer of bioactive molecules to their therapeutic targets is one of the main challenges in cell biology.^1^ In search of strategies for enhancing delivery activity, cell-penetrating peptides (CPPs) have been found to be promising agents with the ability to not only translocate across cell membranes but also transport cargo with a variety of sizes such as small-drug molecules, nucleic acids, and proteins into the cytosol of living cells without substantially damaging the cellular membrane.^2−6^ Among the CPP family, arginine-rich cell-penetrating peptides (ARCPPs) have been extensively investigated since the arginine-rich sequence of the transduction domain of the HIV-1 transactivator of transcription protein was first discovered to efficiently cross the cellular membrane.^7,8^ As a special class of ARCPPs, polyarginines of more than six residues have become well-studied as particularly active CPPs.^9−12^

Despite the significant research effort, the molecular level penetration mechanism of CPPs remains obscure.^13−17^ In general, two major models have been proposed: direct penetration across lipid bilayers as an energy-independent process and endocytosis as an energy-dependent process regulated by cells.^18−20^ It has been found that the uptake efficiency is strongly modulated by experimental conditions, such as temperature and concentration, as well as the types of cells, peptides, and cargo.^20−23^ In contrast to tightly cell-regulated endocytosis, the energy-independent direct translocation mechanism is more interesting from the molecular point of view due to its physically unusual route, which involves the translocation of highly charged CPPs across the hydrophobic core of a lipid bilayer. As this process is energetically unfavorable, it has proven to be difficult to study using conventional computational techniques.^24,25^

A broad interest in ARCPPs has initiated numerous experimental and theoretical studies investigating not only the molecular mechanisms of cell penetration but also the ability of ARCPPs to adsorb to cell membranes, which is a crucial initial step in the translocation process regardless of the subsequent mechanism. Unfortunately, currently available experimental data of peptide adsorption and peptide translocation across biological membranes have been obtained under various conditions, such as different peptide-to-lipid ratios, ionic strengths, or vesicle sizes, and are, therefore, mutually inconsistent. For example, it has been shown by fluorescence measurements at supported lipid bilayers that R_9_ adsorbs at neutral phosphatidylcholine (PC) bilayers with a micromolar dissociation constant, whereas K_9_ does not adsorb at all at low peptide concentrations.^26^ In contrast, in time-dependent fluorescent shift experiments performed on large unilamellar vesicles (LUVs) at high peptide concentrations of R_10_ and K_10_, it has been shown that the differences in the interaction with PC bilayers are negligible.^27^ Similarly, experimental results on the penetration of ARCPPs also differ depending on the experimental conditions. In particular, penetration of ARCPPs across LUVs composed of PC, phosphatidylethanolamine (PE), and negatively charged phosphatidylserine (PS) lipids has been explained by induction of membrane multilamellarity, negative curvature induction, and subsequent formation of a fusion pore similar to membrane fusion induced by calcium.^28^ Importantly, the penetration of ARCPPs has been observed only after the addition of PE and PS lipids but not for LUVs composed solely of zwitterionic PC, which would better represent the extracellular leaflet of the plasma membrane.^28^ Recently, it has been proposed that multilamellarity induction is also responsible for endosomal escape of ARCPPs in cells and late endosome-like LUVs composed of PC and negatively charged bis(monoacylglycero)phosphate lipids.^29,30^ On the other hand, Pujals et al. have shown that induction of positive curvature and lipid phase transition upon ARCPP adsorption at phospholipid bilayers are responsible for pore-free translocation.^31^ In terms of simulations, different studies have predicted different tendencies for polyarginine adsorption onto zwitterionic PC membranes. Robison et al. observed the enrichment of R_9_ on a 1-palmitoyl-2-oleoyl-*sn*-glycero-3-phosphocholine (POPC) surface using an atomistic simulation model,^26^ whereas coarse-grained simulations found no such effect.^32^ Regardless of these differences, no simulation studies have observed the direct permeation of polyarginines, and the free energy barrier extracted for direct R_10_ penetration across the 1,2-dioleoyl-*sn*-glycero-3-phosphocholine (DOPC) bilayer was estimated from biased simulations to be a massive ∼130 kJ/mol.^32^ Other simulation studies have suggested that a potential applied across the membrane could facilitate permeation,^33,34^ yet it is not directly evident how a mechanism based on a potential gradient would discriminate between polyarginines and polylysines. Still, the membrane potential seems to play a role in cells, as demonstrated by a recent experimental study highlighting the role of ion channels in generating permeable pores.^22^ Moreover, very recent experiments with giant unilamellar vesicles (GUVs) composed of neutral PC lipids at very low ARCPP concentrations have actually shown pore-free translocation of ARCPPs,^35,36^ in contrast to previous literature.

Based on the available experimental and simulation data, it is clear that the interaction of peptides with membranes is sensitive to the experimental conditions under which they were measured. Here, we try to understand these differences by systematically examining how the adsorption of cationic peptides onto POPC membranes depends on both peptide and salt concentrations using a combination of molecular dynamics (MD) simulations and fluorescence cross-correlation spectroscopy (FCCS) experiments.^37^ A major methodological issue with classical MD simulations lies in the fact that electronic polarizability is not properly described, resulting in the overestimation of ion–ion interactions, leading to severe artifacts in the description of interactions between charged ions or functional groups in MD simulations.^38^ In order to account for electronic polarizability, it has been proposed that scaling ionic charges by a factor of 0.75 accounts for the missing electronic polarizability in a mean-field manner.^39^ This approach, denoted as “Electronic Continuum Correction” (ECC), has been shown to improve the description of adsorption of ions onto lipid bilayers, as well as the interactions among amino acids.^40−43^ Indeed, the CHARMM36-based ProsECCo lipid and protein models used here rely on the scaled charges. The ProsECCo models provide a realistic structural response of lipid head groups to ion adsorption, while the osmotic coefficient measurements on amino acids also show improved agreement with the experiment as compared to the respective full-charge CHARMM36 models. With ProsECCo, there are regular adsorption and desorption events of ions and peptides to and from a POPC membrane surface so that the system behavior can be studied by unbiased MD simulations at the microsecond time scale. This benefit renders MD simulations with ProsECCo perfectly suited for the efficient probing of different conditions in the system, particularly a wide range of peptide concentrations and ionic strengths. In concert, dual-color FCCS experiments were chosen due to their high sensitivity, low amounts of sample, and short measurement times. In contrast to fluorescent correlation experiments (FCS), they also allow for a more precise determination of binding constants since they do not rely solely on the change in diffusion coefficient but rather on the correlative motion between fluorescently labeled peptides and liposomes, which eliminates the unwanted experimental problems occurring during peptide aggregation at membranes.^37^ Importantly, it should be stressed that the R_9_/K_9_ adsorption measurements in the current experiments use liposomes instead of supported lipid bilayers used in our previous work.^26^ This choice eliminates any support–lipid interactions that might cause pore formation^44^ and ensures that the model system has a similar size and thus average curvature as a cell.

The plasma membrane consists of hundreds of lipid species distributed asymmetrically between its two leaflets.^45^ However, the extracellular leaflet of the plasma membrane contains ∼93% of lipids with a zwitterionic phosphatidylcholine (PC) headgroup, together with small amounts of phosphatidylserine, phosphatidylinositol, and ceramides.^45^ As an initial step in their penetration process, CPPs need to adsorb to this surface made mainly of PC head groups. Thus, in this work, we employed the POPC bilayer as the simplest model for the extracellular leaflet of the plasma membrane to get more insight into the first stage of the translocation mechanism using MD simulations. Our choice of POPC is also supported by the fact that the majority of acyl chains in the lipids of the extracellular leaflet contain either one or no double bonds.^45^ As model peptides, we used nona-arginines (R_9_) and nonalysines (K_9_) since this peptide length is optimal regarding translocation efficiency across lipid membranes.^46^ We also use different concentrations of NaCl and CaCl_2_, thus covering a large number of possible experimental conditions. We accompanied the simulations by FCCS experiments performed at varying peptide concentrations in different ionic solutions. Finally, MD simulations employing ECC give a realistic description of peptide binding, providing a detailed picture of the adsorption of positively charged peptides to neutral PC bilayers and also explaining the differences observed in the available experimental data.

## Computational Methods

### Unbiased Molecular Dynamics Simulations

Atomistic molecular dynamics (MD) simulations were employed to systematically investigate the membrane interactions of nona-arginines (R_9_) and nonalysines (K_9_) in aqueous solutions of either NaCl or CaCl_2_. In order to investigate the influence of both peptide and salt concentrations on the binding, a total of 110 simulations were performed for the varying concentrations of R_9_ or K_9_ peptides (0.007–0.056 m, i.e., Arg/Lys amino acid concentration of 0.063–0.504 m) and NaCl or CaCl_2_ salts (0–1.065 m). The details of all studied systems are summarized in Table S1 in the Supporting Information (SI). The peptides (2 to 16 molecules corresponding to the concentrations above) were initially equally distributed on both sides of the POPC bilayer and relatively close to the interface. Chloride counterions were added to neutralize the systems. The simulation boxes of all studied systems contained 15 856 water molecules. The membrane bilayer consisted of 100 1-palmitoyl-2-oleoyl-*sn*-glycero-3-phosphocholine (POPC) lipids in each leaflet.

In this work, the CHARMM36-based ProsECCo models were applied for lipids and proteins,^42,43,47,48^ and the ion parameters also followed the electronic continuum correction (ECC) approach.^49−51^ In this approach, charges are down-scaled to effectively capture electronic screening effects in a physically justified yet computationally efficient meanfield manner.^40,41,52,53^ ProsECCo, and ECC in general, aim at fixing the common overbinding problems between charged moieties, such as the cationic peptides, salt, and zwitterionic lipid head groups in this work. This is an alternative to the commonly used NBFIX approach that includes specific pairwise terms to the Lennard-Jones potential to prevent such excessive interactions.^48^ While NBFIX has been successful in curing specific excessive charge–charge interaction issues with classical force fields, it is based on fitting empirical observations without physical basis and needs to be parametrized exclusively for each pair of atom types. Here, we used the ProsECCo approach and disabled all the NBFIX corrections, which should capture correctly the most relevant interactions in this work. Specifically, the partial charges in the ProsECCo models, namely, those of the phosphate and choline groups of POPC and the charged groups of arginine and lysine (both the termini and the side chains), were adjusted so that the total charge of each of these groups was scaled down from ±1 to ±0.75. All used topologies are available in Gitlab at https://gitlab.com/sparkly/prosecco/prosECCo75. Lennard-Jones parameters and bonded interactions for lipids and peptides were taken from CHARMM36. All the systems were solvated with the CHARMM-specific TIP3P (“TIPS3P”) water.^54,55^

Buffered Verlet lists were used to track atomic neighbors.^56^ A cutoff of 1.2 nm was used for the Lennard-Jones potential, and the forces were switched to zero between 1.0 and 1.2 nm. The smooth particle mesh Ewald^57,58^ was used for long-range electrostatics. The systems were equilibrated using the Berendsen thermostat and barostat.^59^ For production runs, we used the Parrinello–Rahman barostat^60^ with semi-isotropic pressure coupling and a 1 bar reference pressure, as well as the Nosé–Hoover thermostat^61,62^ with a target temperature of 310 K. The time constants for coupling were set to 5 and 1 ps for the barostat and the thermostat, respectively. Lipid molecules, peptides, and solvent (water and ions) were coupled separately to the thermostats. All covalent bonds in peptides and lipids involving hydrogens were constrained using the P-LINCS algorithm,^63,64^ whereas the SETTLE algorithm^65^ was used for water molecules. MD simulations were performed for 2 μs using the GROMACS package.^66,67^ The time step in all the simulations was set to 2 fs.

The production trajectories were analyzed by in-house Python scripts in conjunction with MDAnalysis library.^68^ The first 500 ns of every trajectory was omitted from the analyses.

### Well-Tempered Metadynamics

The well-tempered metadynamics technique^69^ was employed to directly assess the adsorption free energy of polypeptides at a POPC bilayer. Six systems were modeled, each having either two nona-arginines or two nonalysines solvated in 1.065 m NaCl, 1.065 m CaCl_2_, or salt-free aqueous solution. The two collective variables were the *z*-components of the center-of-mass distances between the membrane and each polypeptide in the system. The selection of the collective variables, together with the system composition, enabled probing the adsorption of one or two polypeptides, which is hardly accessible from unbiased simulations due to rare or zero occurrence of desorption events. Each extracted two-dimensional energy profile was symmetrized along the identity line and then shifted to zero at the bulk value; that is, the lowest free energy when both peptides were further than 4 nm from the membrane was set to zero. To enhance the sampling and convergence, the initial position of the polypeptides was chosen to be close to one of the lipid leaflets. Each collective variable was restrained by a harmonic potential with a force constant of 50 000 and 10 000 kJ mol^–1^ nm^–2^ acting at distances larger than 5.2 and 5.5 nm for salt-free and concentrated solutions, respectively. This prevented the adsorption of either peptide to another leaflet. The initial height of the Gaussians was set to 1 kJ/mol, and their width was 0.1 nm. The bias factor was equal to 10. The total length of the simulations was 1.5 and 3 μs for all K_9_- and R_9_-containing systems, respectively. The Gaussians were added every 1 ps. Aside from the applied bias, the simulation protocol was otherwise identical to the protocol used for unbiased simulations. The metadynamics simulations were performed in GROMACS software with PLUMED plugin.^70^

## Experimental Methods

### Materials

POPC (1-palmitoyl-2-oleoyl-*sn*-glycero-3-phosphocholine), DOPE (1,2-dioleoyl-*sn*-glycero-3-phosphoethanolamine) lipids labeled with ATTO 488 (DOPE-ATTO 488), and 1,2-dioleoyl-*sn*-glycero-3-phosphoethanolamine labeled with ATTO 633 (DOPE-ATTO 633), were purchased from Avanti Polar Lipids (Alabaster, AL, USA). 4-(2-Hydroxyethyl)-piperazine-1-ethanesulfonic acid sodium salt (HEPES), sodium and calcium chloride, sucrose, and 50 nM Whatman Nuclepore Track-Etched Membranes were purchased from Sigma-Aldrich (St. Louis, MO) with a purity of 99%. The μ-Slide 8 well chambers with ibiTreat bottom were purchased from ibidi GmbH (Gräfelfing, Germany). H-(Lys)_9_-OH (or K_9_), H-(Arg)_9_-OH (or R_9_), 5(6)-carboxy-2′,7′-difluorofluorescein-(Lys)_9_-OH (or OregonGreen488-K_9_), and 5(6)-carboxy-2′,7′-difluorofluorescein-(Arg)_9_-OH (or OregonGreen488-R_9_) were synthesized by solid-phase peptide synthesis (SPPS) on a PS3 peptide synthesizer (Gyros Protein Technologies, USA) using standard Fmoc chemistry protocols, 2-(1*H*-benzotriazol-1-yl)-1,1,3,3-tetramethyluronium hexafluorophosphate (HBTU) coupling reagents, and 2-chlorotrityl chloride resin support (0.1 mmol scale, 10 equiv amino acid excess). In the case of peptides labeled with fluorescent dyes, the 5(6)-carboxy-2′,7′-difluorofluorescein^71^ was attached at the last coupling step. Side chain protected peptides were cleaved off the resin with a mixture of acetic acid/2,2,2-trifluoroethanol/dichloromethane (1:1:3) for 2 h at room temperature. The resin was filtered off, reagents and solvents were evaporated to dryness, and residues were treated with a mixture of water/trifluoroacetic acid/triisopropylsilane (95:2.5:2.5). The cleaved and deprotected peptides were lyophilized and purified by RP HPLC (Vydac 218TP101522 column) using methanol and water with 0.05% TFA as solvents. The purity was assessed by analytical RP-HPLC (Vydac 218TP54 column) and LC/MS (Agilent Technologies 6230 ToF LC/MS). The mass was confirmed by MALDI-ToF MS: H-(Lys)_9_-OH [M + H]^+^ 1171.9; H-(Arg)_9_-OH [M + H]^+^ 1423.9; OregonGreen488-K_9_ [M + H]^+^ 1565.9; OregonGreen488-R_9_ [M + H]^+^ 1817.9

### Liposome Preparation

Large unilamellar vesicles (LUVs) were prepared by mixing POPC and DOPE-ATTO 633 at the lipid to dye ratio of 4000:1. Calculated amounts of lipids were put into a glass tube and evaporated with nitrogen. To remove any organic solvent, the samples were put under vacuum overnight. Buffer containing 10 mM HEPES was added to form multilamellar vesicles (MLVs), together with the appropriate concentrations of NaCl and CaCl_2_ when needed. MLVs were extruded through 50 nM Whatman Nuclepore Track-Etched Membranes mounted in a mini-extruder (Avestin, Ottawa, ON, Canada) fitted with two 0.5 mL Hamilton gastight syringes (Hamilton, Reno, NV). The sizes of the resulting LUVs were checked by dynamic light scattering (DLS) with Zetasizer Nano ZS (Malvern Instruments Ltd.) containing a He–Ne laser (532 nm) and an avalanche photodiode detector (APD). The concentration of LUVs used in the experiments is 0.5 mM. Imaging chambers (Ibidi Uncoated) were incubated sequentially with 0.1 mg/mL BSA (Sigma) dissolved in Milli-Q water prior to the addition of the sample.

### Set-Up for Fluorescence Cross-Correlation Spectroscopy (FCCS)

The microscope, Olympus IX71, body consists of standard confocal parts: a 635 dichroic mirror (Chroma, USA), a water immersion objective (UPLSAPO 60× , Olympus, Japan), a 3D sample scanning stage (PI Mars XYZ NanoPositioner, PI, Germany), and a 50 μm pinhole. The detection unit consists of 697/58 and 525/50 nm emission filters (Chroma, USA) and a pair of single photon avalanche diodes (PDM, MPD, Italy). Samples were excited by 470 nm (LDHP-C-470, Picoquant, Germany) and 635 nm (LDH-D-C-635, Picoquant, Germany) lasers operated at 10 MHz repetition rate each and pulsed alternatively. The laser power was kept between 1 and 4 μW.

### Measurements of Peptide Binding Curves Using FCCS

ATTO 488 and Alexa Fluor 647 were used to determine the effective detection volume sizes for blue and red laser, respectively. The effective cross-correlation detection volume, *V*_eff,x_, was calculated from
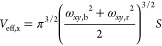
1In this equation, *S* is the structural parameter,^72^ and ω_*xy*,b_ and ω_*xy*,r_ are the focus radii of blue and red lasers, respectively. ω_*xy*,b_ and ω_*xy*,r_ were determined using the tabulated values of the diffusion coefficients (*D*) for ATTO 488 and Alexa Fluor 647, respectively, and measured diffusion time of the dyes. Before any measurement, double-labeled LUVs (POPC with DOPE-ATTO 633 and DOPE-ATTO 488) were used to determine the maximum achievable cross-correlation amplitude characterizing the microscope alignment. The concentration of lipids was kept at 0.6 mM. Unlabeled R_9_ and R_9_ labeled with Oregon Green 488 were mixed at 10:1 molar ratio. Different amounts of this mixture were incubated with LUVs for 10 min before any measurement. Each point was measured for at least 1 min.

### Analysis of FCCS Data

The analysis is based on calculating a so-called cross-correlation function, *G*_x_(τ) defined as
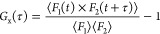
2This function correlates the intensity in one channel at time *t* (*F*_1_(*t*)) with the intensity in the other channel at time τ later (*F*_2_(*t* + τ)). In this work, we correlated the intensity in the blue channel (corresponding to fluorescently labeled R_9_/K_9_ peptide) with the intensity in the red channel (corresponding to fluorescently labeled vesicles). In this way, we could follow binding of R_9_/K_9_ peptides to lipid vesicles. Whereas a positive cross-correlation amplitude indicates binding, zero cross-correlation amplitude indicates that most of peptide molecules are left in the solution without interacting with the vesicles. FCCS may also be used to calculate the dissociation constant of binding. This analysis is based on the determination of the actual number of bound peptides per LUV, ⟨NP⟩, and the concentration of free peptide in the solution, *C*_P_^free^, from FCCS data. In case of noncooperative binding, *C*_P_^free^ and ⟨NP⟩ are related through a Langmuir isotherm. This reads
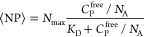
3where *K*_D_ is the dissociation constant, *N*_max_ is the maximum number of binding sites per LUV, and *N*_A_ is Avogadro’s number. Thus, *K*_D_ and *N*_max_ can be determined by fitting experimental FCCS data to eq 3. ⟨NP⟩ can be determined from the known cross-correlation amplitude (*G*_x_^0^, *V*_eff,x_) and the total peptide concentration, *C*_P_^0^, as in ref (37):
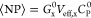
4Similarly, *C*_p_^free^ is calculated as in ref (37).
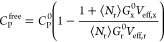
5where ⟨*N*_r_⟩ is the number of lipid dye molecules per LUV, while *G*_r_^0^ is the red autocorrelation amplitude determined using Origin software, and *V*_eff,r_ is the effective detection volume of the red laser.

## Results and Discussion

### Polyarginine and Polylysine Display Qualitatively Different Adsorption Tendencies

We performed an extensive set of atomistic simulations of a POPC bilayer with varying numbers of R_9_ and K_9_ peptides either in pure water or in solutions containing NaCl or CaCl_2_. The free energies of adsorption of R_9_ and K_9_ peptides onto a POPC bilayer were computed from symmetrized averaged number density profiles of peptides along the *z* direction from the bulk solution to the membrane surface using the expression:
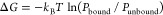
6Here, *k*_B_ is the Boltzmann constant, *T* is absolute temperature, and *P*_bound_ and *P*_unbound_ were defined as probabilities of the peptide being in bound and unbound regions with respect to the membrane surface, respectively. These probabilities are obtained by integrating the normalized peptide number density distribution, ρ, over the relevant region, namely,
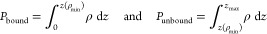
7where the cutoff distance *z*(ρ_min_) is the *z* coordinate where the density distribution reaches its minimum value ρ, *z* = 0 refers to the membrane center, and *z*_max_ is the maximum value of the *z* coordinate in the simulation box. For density profiles with no specific binding peaks (usually the case for K_9_), the cutoff value was chosen as 3.5 nm, which is the average calculated over the other *z*(ρ_min_) values.

**Figure 1:**
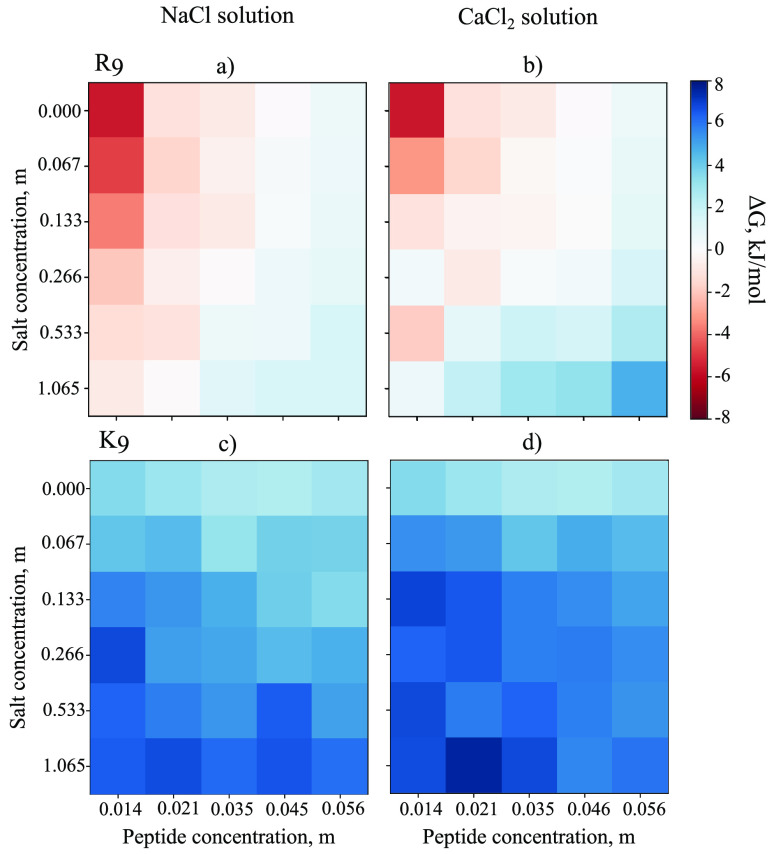
Free energy Δ*G* (kJ mol^–1^) of the binding of R_9_ (top row) or K_9_ (bottom row) peptides to the POPC membrane surface with various peptide and salt concentrations in the solution.

Free energy differences, Δ*G*, calculated from all of our 110 simulations for R_9_ and K_9_ are presented in Figure 1 as heat maps of two variables, the nominal peptide and salt concentrations. Looking first at the systems without salt (top rows in the panels in Figure 1), it is evident that R_9_ preferentially binds to POPC at peptide concentrations up to 0.035 m (Δ*G* < 0). At the lowest R_9_ concentration, with four peptides present in the system, the binding is the most stable at around −6 kJ/mol, whereas the value rapidly decreases upon increasing R_9_ concentration. Notably, the Δ*G* value changes sign at 0.046 m, indicating that at higher peptide concentrations, the R_9_ peptides prefer to remain in the solution rather than adsorb onto the membrane. This behavior is also demonstrated by the top row in Figure S1, which shows the effect of peptide concentration on their density profiles. At higher peptide concentrations, many of the peptides reside in the solution for most of the simulation time. This observation can be explained by two factors: peptide adsorption saturation at the membrane surface and an increase in ionic strength in the bulk due to the presence of positively charged peptides. Such behavior is expected and provides the typical Langmuir-like isotherm at low salt concentrations. Still, the free energy values reported here for different peptide concentrations are useful in describing the peptide populations in the solution and on the POPC surface, with implications for the interpretation of experimental data.

Comparing next the behavior of R_9_ (top two panels in Figure 1) with that of K_9_ (bottom two panels in Figure 1), it is evident that the two peptides display opposite binding behavior. Indeed, it is clear that K_9_ peptides do not adsorb onto the POPC surface at any peptide concentration, with positive values of Δ*G* ranging from 2.9 to 7.5 kJ/mol. These low binding affinities of K_9_ are also demonstrated by the density profiles (see Figure S4), where density at binding sites (lipid headgroup position) is depleted compared to the bulk phase. Several experimental and theoretical investigations have reported similar findings.^26,73,74^ The difference in behavior between R_9_ and K_9_ has been previously reported as a result of the differences in their molecular structures. Polyarginine consists of guanidinium moieties, which can strongly bind to phosphate by electrostatic attraction and hydrogen bonding, whereas ammonium groups in polylysine exhibit poor interaction with phosphate.^26,75^ Furthermore, a recent study using SFG-VS experiments^17^ also showed the ability of arginine amino acid to reach deeper into the bilayer compared to lysine via its interactions with carbonyl groups.

Moreover, the peptide concentration appears to have an opposite effect on the membrane binding of K_9_ than what was observed for R_9_. In other words, the increasing amount of K_9_ peptides caused a decrease of Δ*G* values. However, this trend follows from the simple fact that interpeptide repulsion at higher concentrations slightly pushes peptides to the vicinity of the membrane surface, thus leading to an increased density in that region even without specific binding. This is corroborated by the density profiles shown in Figure S4.

While our unbiased simulations are performed using state-of-the-art computer resources, sampling the binding equilibrium is challenging with only a few peptides, that is, when the binding is most favorable. We calculated the minimum distances of each peptide from the nitrogen atoms of the POPC choline groups, and these data are shown for selected systems in Figure S5. With larger peptide concentrations, the peptides regularly switch between the bound and unbound states, indicating that the free energy estimates derived from density profiles are reliable. However, in the simulations containing only four peptides, one of them might never dissociate from the membrane during the simulation time. Thus, it is entirely possible that equilibrium would only be sampled at significantly longer simulation times. This indicates that for low peptide concentrations, our free energy estimates serve as lower bounds for the binding free energy.

**Figure 2:**
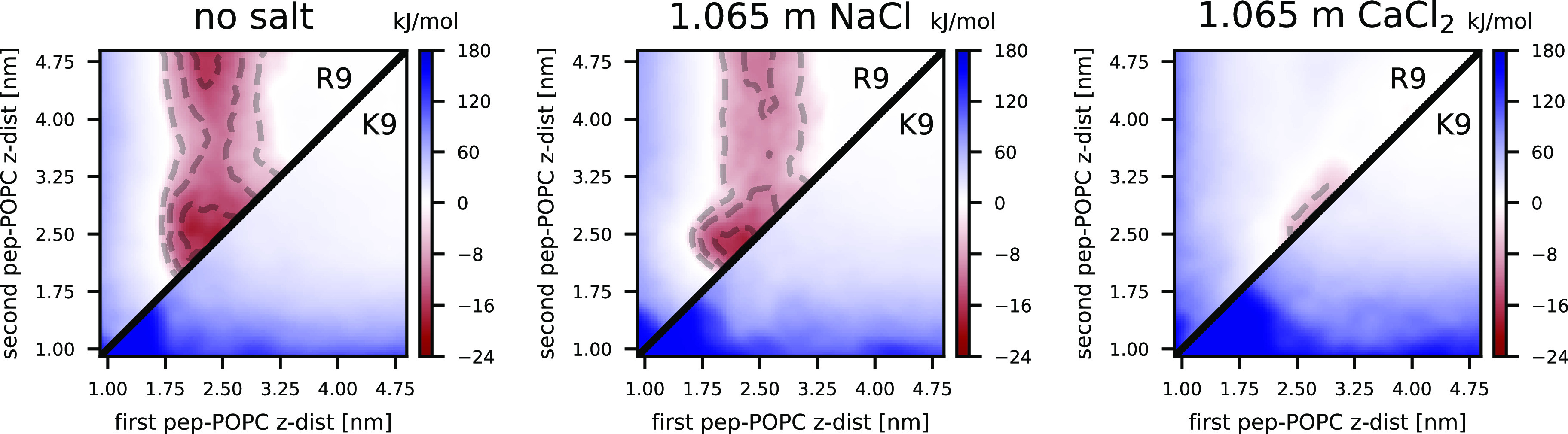
Resulting postprocessed two-dimensional free energy profiles for R_9_ (top-left triangle) or K_9_ (bottom-right triangle) peptides adsorbed to the POPC membrane under different ionic concentration conditions. The contours are added for −5, −10, and −15 kJ/mol. The evolution of the total adsorption free energy, demonstrating convergence, is shown in Figure S3.

Fortunately, in this low-concentration regime, the system behavior can be described by a couple of intuitive reaction coordinates and thus probed using biased simulation approaches. We chose to perform well-tempered metadynamics simulations to probe the binding of two R_9_ or K_9_ peptides to the same POPC leaflet. As demonstrated in Figure S3, the metadynamics simulations indeed converge well within the simulation time. The free energy surfaces extracted from these simulations in the absence of salt are shown in the leftmost panel of Figure 2 and confirm that for R_9_, there is a clear global free energy minimum of approximately −20 kJ/mol at the membrane surface, at around 2.2 nm away from the membrane core. In contrast, no such minimum is observed for K_9_. Instead, the most favorable positioning for K_9_ is in the aqueous phase, as far from the POPC surface as allowed by our simulation setup.

Having convincingly demonstrated the difference between R_9_ and K_9_ adsorption onto the zwitterionic membranes using our implicitly polarizable simulation model, we verified these findings experimentally. To this end, we performed fluorescence cross-correlation spectroscopy (FCCS) measurements for systems where POPC vesicles were exposed to either R_9_ or K_9_, without the presence of salt ions. In these experiments, 10% of R_9_ and K_9_ are labeled with Oregon Green 488 fluorescent label, with the zwitterionic POPC membrane containing fluorescently labeled lipids (see Experimental Methods).

**Figure 3:**
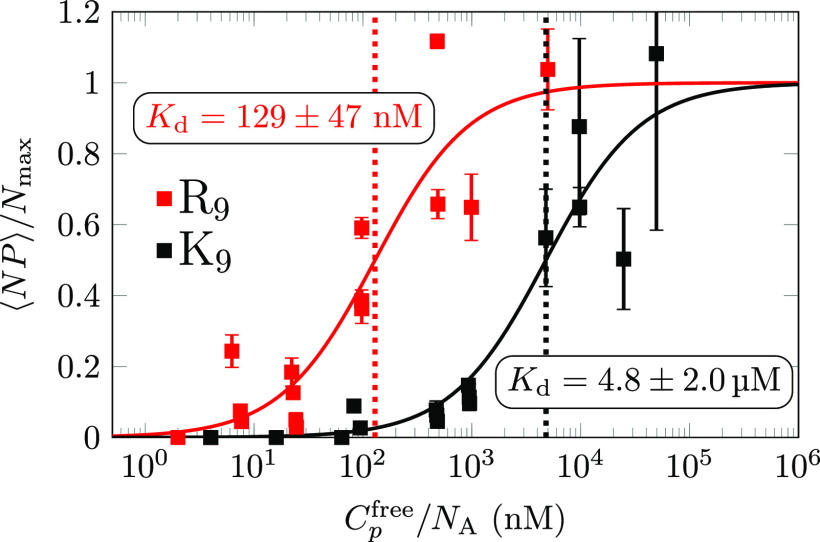
Experimentally determined Langmuir binding isotherms for R_9_ (red squares) and K_9_ (black squares) peptides and the corresponding fits (solid lines; assuming independent sites and noncooperative binding) relating the number of binding sites for the peptide per LUV and the concentration of free peptide in the bulk. The data are normalized so that eq 3 converges to 1 at large concentrations.

Figure 3 shows the experimentally determined Langmuir binding isotherms for labeled R_9_ and K_9_. The Langmuir fit assumes independent binding sites and no interactions among the peptides (*i.e*., noncooperative binding). The inspection of the data and the analysis of the corresponding fits show that R_9_ binds almost 2 orders of magnitude stronger to POPC with *K*_d_ of 129 ± 47 nM in contrast to K_9_, which adsorbs weaker with *K*_d_ of only 4.8 ± 2.0 μM. The adsorption of R_9_ is tighter than the previously determined *K*_d_ using fluorescence binding assays of unlabeled peptides where this value was determined to be 70 ± 19 μM.^26^ In addition to small experimental setup differences (see below for details), this could be a consequence of the fact that adsorption to the surface of supported lipid bilayers^26^ is, in general, mechanistically also slightly different than adsorption onto free-standing LUVs used in this work. In line with this, we actually detected the adsorption of labeled K_9_ in FCCS experiments in contrast to present MD simulations and fluorescence binding assays performed using supported lipid bilayers and unlabeled peptides.

**Figure 4:**
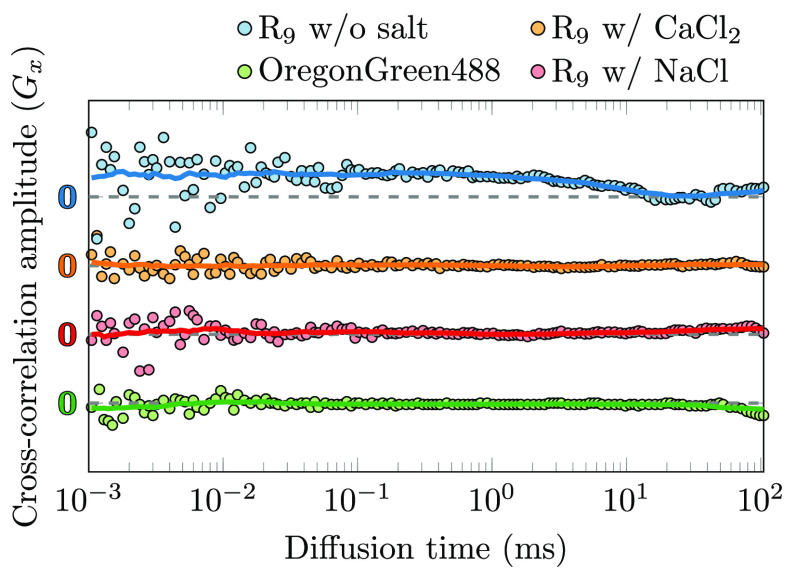
Calculated cross-correlation functions shown for R_9_ peptides (500 nM concentration) in the absence or presence of 100 mM concentration of NaCl or CaCl_2_. Also, a cross-correlation function for free Oregon Green 488 dye is shown. The curves are vertically shifted for clarity. The markers show measured data points, whereas the lines show running averages over 20 data points. The concentration of Oregon Green 488 in all correlations is 50 nM. A positive cross-correlation indicates binding to the membrane, whereas zero cross-correlation points to nondetectable binding.

To clarify the possible role of the attached Oregon Green 488 label itself in the peptide-binding strength, we performed control binding FCCS experiments with free Oregon Green 488 fluorophore in the presence of POPC vesicles. Here, as shown in Figure 4, no positive cross-correlation was detected. However, it cannot be completely excluded that K_9_ attached to the Oregon Green 488 probe increases the possibility of labeled K_9_ binding to POPC.^76^ In addition, we should also mention that the experiments in ref (26) and the ones performed in this work are also not fully comparable. Specifically, we used a 10 mM HEPES buffer in contrast to the work presented in ref (26), where 10 mM phosphate-buffered saline (PBS) and 150 mM NaCl were employed in the experiments. The use of different buffers in the reported experiments can also affect the adsorption of ions and peptides at membranes, especially when the buffer concentrations are comparable (or even higher, as in our case) to the peptide concentrations used in binding experiments. Indeed, some commonly used buffers can even alter the physical properties of the bilayers.^77−79^ A detailed analysis of cross-correlation functions upon the addition of salts and increase of ionic strength is presented in the next section.

Taking into account all possible experimental differences between the different experimental setups, the most important difference in binding of R_9_ vs K_9_ onto POPC is still that R_9_ binds much tighter than K_9_, which is in full agreement with the results reported in ref (26). Our data definitely confirm the binding of arginine-rich peptides to zwitterionic POPC bilayers, which may serve as a key point in explaining why arginine-rich peptides easily penetrate POPC vesicles as well as cellular membranes, whereas lysine-rich peptides do not.

### The Ionic Strength of the Solution Qualitatively Alters the Binding Behavior

Having established that R_9_ and K_9_ show very different binding affinities to POPC membranes and that this binding heavily depends on the peptide concentration, we moved on to the effect of salts. As seen by comparing the individual rows in Figure 1a,b, the presence of salts significantly affects the adsorption of R_9_ molecules onto a POPC membrane. Particularly, for a given R_9_ concentration, increasing salt concentration increases the Δ*G* values; thus, the more salt that is present in the system, the weaker the membrane–peptide interaction becomes. Salt even alters the qualitative binding behavior. For example, at a peptide concentration of 0.035 m and low salt concentrations, peptide binding is favorable (Δ*G* < 0), but at higher salt concentrations, Δ*G* becomes increasingly positive, suggesting that the peptides rather remain in the solution. Density profiles for individual peptides with different salt solutions are presented in Figures S1 and S4. These profiles reveal that the decrease of the apparent averaged free energy of R_9_ adsorption is not due to a decrease of individual peptide adsorption free energies. In contrast, the decrease results from the combined effect where several peptides remain strongly bound at POPC, while the rest remain in the bulk and cannot adsorb anymore to the saturated POPC surface. The same effect of ionic strength is observed for K_9_, Figure 1c,d; Δ*G* increases with increasing salt concentration.

The R_9_–membrane interactions depend on not only salt concentration but also the nature of the salt. The Δ*G* values for R_9_–POPC systems embedded in CaCl_2_ solutions are higher than those in NaCl solutions at the same concentrations. However, as Ca^2+^ is a divalent ion, the comparison of salt concentrations is somewhat misleading. By comparing the peptide binding in NaCl and CaCl_2_ solutions having the same charge concentration (compare each row in Figure 1a with a row one step higher in Figure 1b), our results still show that the Δ*G* values are higher in the CaCl_2_ solution than in the NaCl solution. This implies that Ca^2+^ ions more efficiently decrease the binding affinity of R_9_ to a POPC bilayer than Na^+^ ions. A similar decrease in binding affinity was observed in an experimental study of R_8_–DOPC (or DOPC/DOPG) GUV interactions with Na^+^.^80^ The stronger effect of CaCl_2_ compared to NaCl could result from two factors. First, the screening of electrostatic interactions is given by ionic strength, which scales as *z*^2^ for an ion with a charge of *z*. Thus, at the same charge density, CaCl_2_ provides a stronger screening effect than NaCl, thus reducing more efficiently the attraction between the POPC head groups and R_9_. Second, sodium and calcium ions may also adsorb onto the headgroup region of zwitterionic POPC membranes and thereby introduce a positively charged surface that will repel the cationic arginine residues, in turn reducing the binding affinity of R_9_. The magnitude of this effect is dictated by the total surface charge, which depends on the ion charge and adsorption preferences.

Our findings are somewhat different from those observed in atomistic simulations using the Slipids lipid model in combination with the Amber ff99SB protein model.^26^ The authors of this study proposed the preferential binding of R_9_ onto POPC surfaces even at peptide concentrations of ∼0.028 m or ∼0.056 m in the presence of 0.150 M NaCl. Moreover, the effect of an increased peptide concentration was minor. A logical explanation for this discrepancy is the different force fields used. Since few quantitative experimental data on the adsorption of cationic peptides are available, it is not easy to judge which model is more accurate. However, the Slipids force field is known to exaggerate the adsorption of positive ions onto PC membranes.^53^ Therefore, the stronger adsorption of cationic R_9_ in the Slipids model is not surprising. The ProsECCo model, in turn, is designed to resolve this somewhat unrealistic electrostatic binding.

This brings us to the question of whether our ProsECCo model based on scaled charges may predict too little R_9_ adsorption onto PC membranes. To ensure that this is not the case, we performed a pair of simulations with a POPC membrane and two R_9_ peptides with both CHARMM36 and ProsECCo models. As shown in Figure S2 in the SI, the binding affinity for these models is fairly similar. However, peptides penetrate deeper into the membrane in the case of CHARMM36, with the density peaking at ca. 2 nm from the center of mass of the bilayer. With ProsECCo, the density reaches a maximum at ca. 2.3 nm, and the distributions are slightly broader, indicating a less specific binding mode. The more specific interactions in the full-charge CHARMM36 model are visible in the radial distribution functions between the arginine side chain (namely, guanidinium carbon atom) and POPC choline nitrogen atom or POPC phosphate atom, respectively (see the bottom panel in Figure S2). This suggests that while improving the description of ion–lipid interactions, ProsECCo does not lead to qualitative differences in lipid–peptide interactions.

For additional validation, we repeated our metadynamics simulations with 1.065 m of either NaCl or CaCl_2_. As demonstrated in the middle and rightmost panels of Figure 2, the K_9_ peptides do not prefer to adsorb to POPC membrane under any conditions, whereas the adsorption of R_9_ is regulated by the ionic concentration. Our metadynamics simulations cover the extreme cases of the unbiased simulations, that is, the highest salt concentration and the case of no salt. The free energy landscapes clearly show how the adsorption free energy increases from approximately −20 kJ/mol (no salt) to approximately −15 kJ/mol and approximately −10 kJ/mol in 1.065 m NaCl and 1.065 m CaCl_2_ solutions, respectively. Interestingly, in the case of R_9_ in CaCl_2_ solution, the binding mode when only one peptide is adsorbed is not energetically favorable compared to when both peptides are adsorbed, which indicates that dual R_9_ adsorption at POPC is possibly facilitated by R_9_–R_9_ interactions in solution^81^ or at the POPC bilayer.^26^

We also performed FCCS experiments to confirm the effect of salts on R_9_ adsorption onto a POPC membrane. Figure 4 shows the cross-correlation functions for labeled R_9_ at POPC in the absence and after addition of Na^+^ or Ca^2+^ ions. Here, 100 mM NaCl or CaCl_2_, respectively, were added to a system containing 500 nM R_9_ in 10 mM HEPES solution. Two important points can be deduced from the experiments. First, the cross-correlation is detected for labeled R_9_ in interaction with POPC (blue curve), showing that these labeled peptides indeed adsorb to the zwitterionic PC membranes. Second and more importantly, we clearly see that the addition of Na^+^ or Ca^2+^ induces a complete loss of the cross-correlation (orange and red curves), implying that both Na^+^ and Ca^2+^ ions effectively prevent the adsorption of R_9_ onto the POPC membranes under given experimental conditions. This is in accordance with our MD simulation results, which show a relative decrease of total R_9_ adsorption to POPC upon the increase in ionic strength by either the addition of peptides or the addition of NaCl or CaCl_2_. This further confirms that the adsorption of peptides onto the membranes is strongly altered or can even be inhibited by the addition of salts or a further increase in peptide concentration in the experiments.

### Salt Affects Peptide Binding More Than Peptides Affect Cation Binding

**Figure 5:**
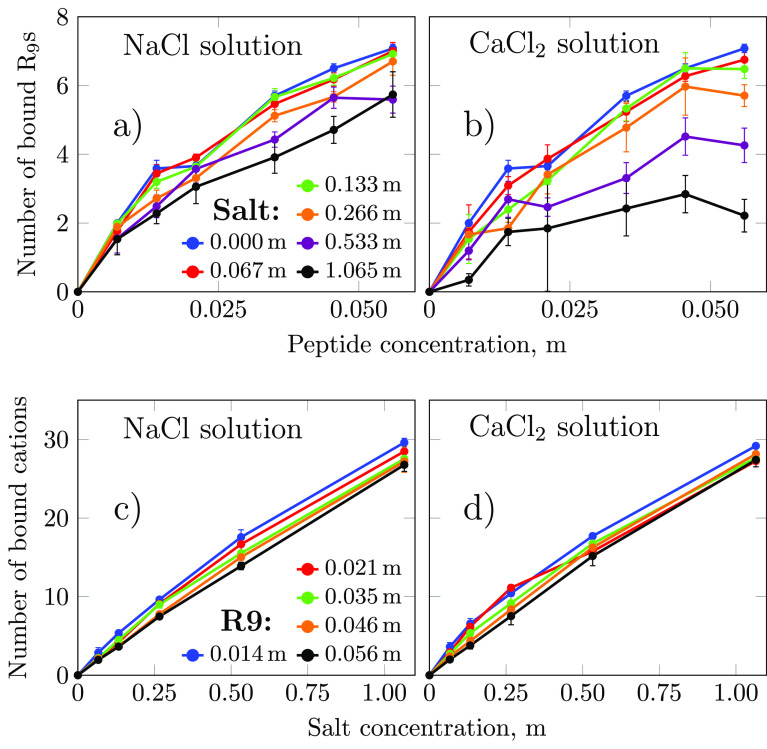
Change in a number of bound R_9_ peptides and salt cations (Na^+^, Ca^2+^) to the membrane surface with respect to their concentrations in solution.

Next, we analyzed the effects of salt on R_9_ binding and *vice versa*. The number of adsorbed R_9_ molecules on the POPC surface for each system was calculated from the corresponding average number density profiles, and the results are displayed in Figure 5a,b. In both NaCl and CaCl_2_ aqueous environments, the number of adsorbed R_9_ molecules decreases with increasing the salt concentration, as we noted previously. However, the degree of adsorption is different between Na^+^ and Ca^2+^ solutions as deduced from the free energy calculations. At the same concentration, Ca^2+^ significantly reduces the adsorption of R_9_ peptides, and the membrane surface is saturated at a peptide concentration of 0.046 m. Moreover, in the case of the highest salt concentration and peptide concentration of 0.056 m, the actual number of membrane adsorbed R_9_ peptide starts to decrease (although still within the error estimate). While Na^+^ only mildly decreases the number of adsorbed R_9_ molecules, the saturation coverage may be reached at a concentration beyond the range studied in this work (0.007–0.056 m). Unlike the strong effect that cation–lipid interaction has on peptide adsorption, the peptide–lipid binding at the interface negligibly impacts Na^+^ or Ca^2+^ adsorption. Apparently, only a slight decrease in the number of bound Na^+^ or Ca^2+^ is seen as the peptide concentration increases (Figure 5c,d). This tendency is partially in line with ref (82), where the adsorption of sodium ions was reported as being independent of the presence of polycations.

Since numerous studies highlighted the important role of positively charged guanidinium moieties (Gdm^+^) in R_9_–phospholipid interactions,^12,26,27^ it is interesting to check the effect of ionic strength on these interactions. Gdm^+^, together with Na^+^ or Ca^2+^, preferentially interacts with the phosphate and choline groups of POPC (see Figure S1), which leads to the competition for the adsorption sites on the POPC bilayer between R_9_ and monatomic cations. In this context, we calculated how many Gdm^+^ moieties are directly bound to the surface when the peptide is bound to the POPC surface. The results are displayed in Table 1. Each adsorbed R_9_ molecule donates on average 2.5–5.9 arginine side chains to bind to the bilayer. Bound R_9_ molecules in NaCl solutions are stabilized by more Gdm^+^ groups than those in CaCl_2_ solutions, and these values tend to decrease with the addition of NaCl or CaCl_2_. This points to the fact that the binding of initially strongly bound peptides (before saturation of POPC bilayer) also tends to slightly decrease as the ionic strength increases in the bulk. Interestingly, we also observe the same trend as more peptides are added into the solutions due to increased ionic strength by adding positively charged peptides. Taken all together, an increase in the concentration of either salts or peptides generally lowers the ability of the peptides to adsorb onto the POPC bilayer, by both diminishing electrostatic interactions of strongly bound peptides and saturating the bilayer surface.

**Table 1:** Number of Guanidinium Groups per Bound R_9_ Buried into the POPC Membrane in the System with Given Peptide and Salt Concentrations [m]^a^

	R_9_ peptide					
salt	0.007	0.014	0.021	0.035	0.046	0.056
0.000	5.9	5.3	5.3	4.9	4.4	4.3
0.067	5.5/5.5	5.3/5.2	5.4/5.1	5.0/4.5	4.7/4.3	4.5/4.3
0.133	5.9/5.9	5.6/4.3	5.2/4.8	4.8/4.7	4.7/4.2	4.2/4.1
0.266	5.8/4.9	5.4/4.6	4.5/4.8	4.5/4.5	4.6/4.4	4.2/4.0
0.533	5.2/4.3	4.9/4.7	4.8/3.5	4.1/3.5	4.4/3.8	3.7/3.3
1.065	5.0/3.0	4.8/3.8	4.4/3.4	3.6/3.0	3.9/3.1	3.7/2.5

a The values to the left correspond to NaCl solutions; the values to the right are shown for CaCl_2_ solutions.

### Structural Changes of the Membrane upon Peptide Adsorption

**Figure 6:**
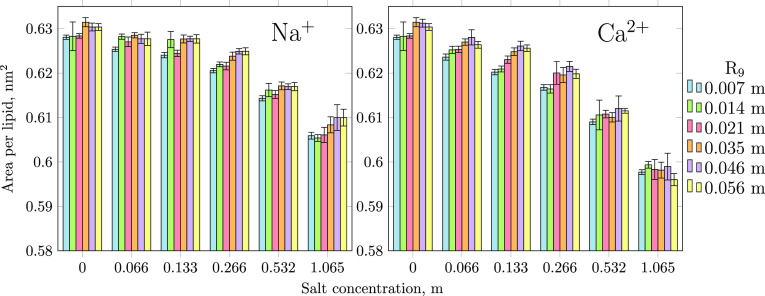
Changes in area per lipid with varying R_9_ and salt (NaCl or CaCl_2_) concentrations.

Finally, we address the influence of peptide and ion adsorption on the area per lipid (APL) of the POPC bilayer. In the case of ions and cationic peptides that do not penetrate to the acyl chain regions, APL alone is a good parameter to describe membrane structure, as it is inversely correlated with membrane thickness and order. The results, shown in Figure 6, demonstrate that both adsorption processes cause changes in APL, but these effects are opposite to each other. In particular, the increase in the number of adsorbed salt ions at the surface significantly reduces APL (Ca^2+^ with its stronger binding affinity leading to a larger APL reduction than Na^+^), which results from the cations fitting between the POPC head groups and bridging them together.^83^ Thus, it seems that cations have a 2-fold effect on the efficiency of R_9_ penetration: they reduce the amount of adsorbed peptides and render the membrane more packed and hence more rigid against the formation of pores, bifurcations, or other membrane defects. In contrast, APL tends to slightly increase as the number of R_9_ molecules adsorbed at the POPC surface increases. This is likely caused by the penetration of R_9_ into the lipid headgroup region followed by the expansion of the membrane due to steric stress imposed by the relatively large R_9_ molecules.

## Conclusions

Unbiased molecular dynamics (MD) simulations, metadynamics simulations, and complementary fluorescence cross-correlation spectroscopy (FCCS) experiments were employed to characterize the interactions of positively charged peptides, polyarginines and polylysines, with zwitterionic POPC membranes at varying concentrations of NaCl or CaCl_2_. MD simulations reveal several important aspects of the peptide–membrane interactions. First, we show by MD simulations that R_9_ peptides have a stronger preference for POPC bilayer compared to K_9_ peptides, which do not adsorb to POPC in any of the studied conditions. This finding agrees with earlier simulations and experiments at supported lipid bilayers^26^ and also serves as an important benchmark for the employed ProsECCo interaction model incorporating polarization by scaled charges.

Second, the apparent peptide adsorption decreases with increasing peptide and salt concentrations. In particular, the average adsorption energy of R_9_ peptides increases from ca. −6 kJ/mol in the case of no added salt and low peptide concentration to +2 kJ/mol in the case of high peptide concentration and the addition of 1.065 m NaCl or +6 kJ/mol upon the addition of 1.065 m CaCl_2_. In the case of K_9_, the peptides do not stably adsorb onto POPC under any conditions. These results are supported by metadynamics simulations, which demonstrated the same binding tendency of the R_9_ and K_9_ peptides to the bilayer. Particularly, K_9_ exhibits low affinity to the membrane bilayer in contrast to R_9_, and the addition of CaCl_2_ results in a larger increase of adsorption free energy than the addition of NaCl. The diminution of peptide adsorption is mostly due to the saturation of the POPC surface and the simultaneous increase of the peptide concentration in the bulk solution. However, the individual R_9_ peptide binding affinity also slightly decreases in solutions of high ionic strength.

Our complementary FCCS experiments also capture the trends observed in the MD results. Specifically, we observe that fluorescently labeled R_9_ binds by almost 2 orders of magnitude tighter than K_9_ to the zwitterionic POPC bilayer with *K*_d_(R_9_) = 129 ± 47 nM/dm^3^ and *K*_d_(K_9_) = 4.8 ± 2.0 μM/dm^3^, respectively. This corresponds to a ∼9 kJ/mol shift in the binding free energy. In the FCCS experiments, K_9_ still demonstrates significant binding, which likely results from the interactions of the fluorescent probe and the membrane. Indeed, previous experiments found no binding of K_9_ to a PC bilayer with a different fluorescence assay.^26^ In our FCCS experiments, the addition of NaCl or CaCl_2_ completely suppresses the fluorescence cross-correlation signal, indicating that the addition of either salt leads to a decrease in peptide binding on average, in agreement with the MD simulations.

Our methodology relies on high-throughput unbiased simulations, which can be used to efficiently scan different conditions on peptide binding. However, their predictions become unreliable in the case of low peptide concentration, where the tight binding of R_9_ prevents the efficient determination of free energies from an equilibrium of bound and unbound peptides. Fortunately, our less efficient metadynamics approach can be adapted here to sample the case where the reaction coordinate is intuitive. Finally, we also used FCCS experiments, which have their own limitations due to uncertainties in the experimental setup and sample preparation, as well as the necessary use of probes. Still, all three approaches provide us with the same trends, indicating that our findings are solid, even if the free energy estimates can be considered qualitative.

Taken together, our simulations and experiments generally show that the increase of peptide or salt concentration, that is, increase of ionic strength in the system, results in diminishing binding of positively charged peptides at the zwitterionic POPC bilayer dominantly due to electrostatic screening induced by additional charge in the bulk. This has important consequences and calls for careful planning of experiments and simulations involving the binding of polycationic peptides to lipid bilayers. The experimental results can greatly depend on the initial conditions, possibly even leading to qualitatively different outcomes in terms of peptide–membrane adsorption. As the cell penetration of polyarginines (and other charged cell-penetrating peptides) involves peptides binding to the membrane as an initial step of the cell translocation mechanism, it is crucial to consider the effects of salt and peptide concentrations in the cell penetration studies as well.

## Supporting Information Available

Table summarizing the components of simulation boxes, additional descriptions of binding of R_9_ and K_9_ to POPC bilayer in terms of density profiles, metadynamics, radial distribution functions, and minimum distance calculations (PDF)
