# Supplementary material for: Ionic Strength and Solution Composition Dictate the Adsorption of Cell-Penetrating Peptides onto Phosphatidylcholine Membranes
Source: Langmuir. 2022 Sep 9;38(37):11284–95. doi: 10.1021/acs.langmuir.2c01435 (PMC9494944; doi:10.1021/acs.langmuir.2c01435)
Supplement: Supplementary file 1 — la2c01435_si_001.pdf [file la2c01435_si_001.pdf]

Supporting Information: Ionic Strength and Solution Composition Dictate the Adsorption of Cell-Penetrating Peptides onto Phosphatidylcholine Membranes

Man Thi Hong Nguyen,[†] Denys Biriukov,[†] Carmelo Tempra,[†] Katarina Baxova,[†] Hector Martinez-Seara,[†] Hüseyin Evci,[‡,¶] Vandana Singh,[‡] Radek Šachl,[‡] Martin Hof,[‡] Pavel Jungwirth,[†] Matti Javanainen,[*,†,§] and Mario Vazdar[*,†,‖]

†*Institute of Organic Chemistry and Biochemistry of the Czech Academy of Sciences, Flemingovo nám. 542/2, CZ-16000 Prague 6, Czech Republic*

‡*J. Heyrovský Institute of Physical Chemistry of the Czech Academy of Sciences, Dolejškova 2155/3, CZ-18223 Prague 8, Czech Republic*

¶*Department of Chemistry, Faculty of Science, University of South Bohemia in Ceske Budejovice, 370 05 Ceske Budejovice, Czech Republic.*

§*Institute of Biotechnology, University of Helsinki, FI-00014 University of Helsinki, Finland*

‖*Department of Mathematics, University of Chemistry and Technology, 166 28 Prague, Czech Republic*

E-mail: matti.javanainen@helsinki.fi; mario.vazdar@vscht.cz

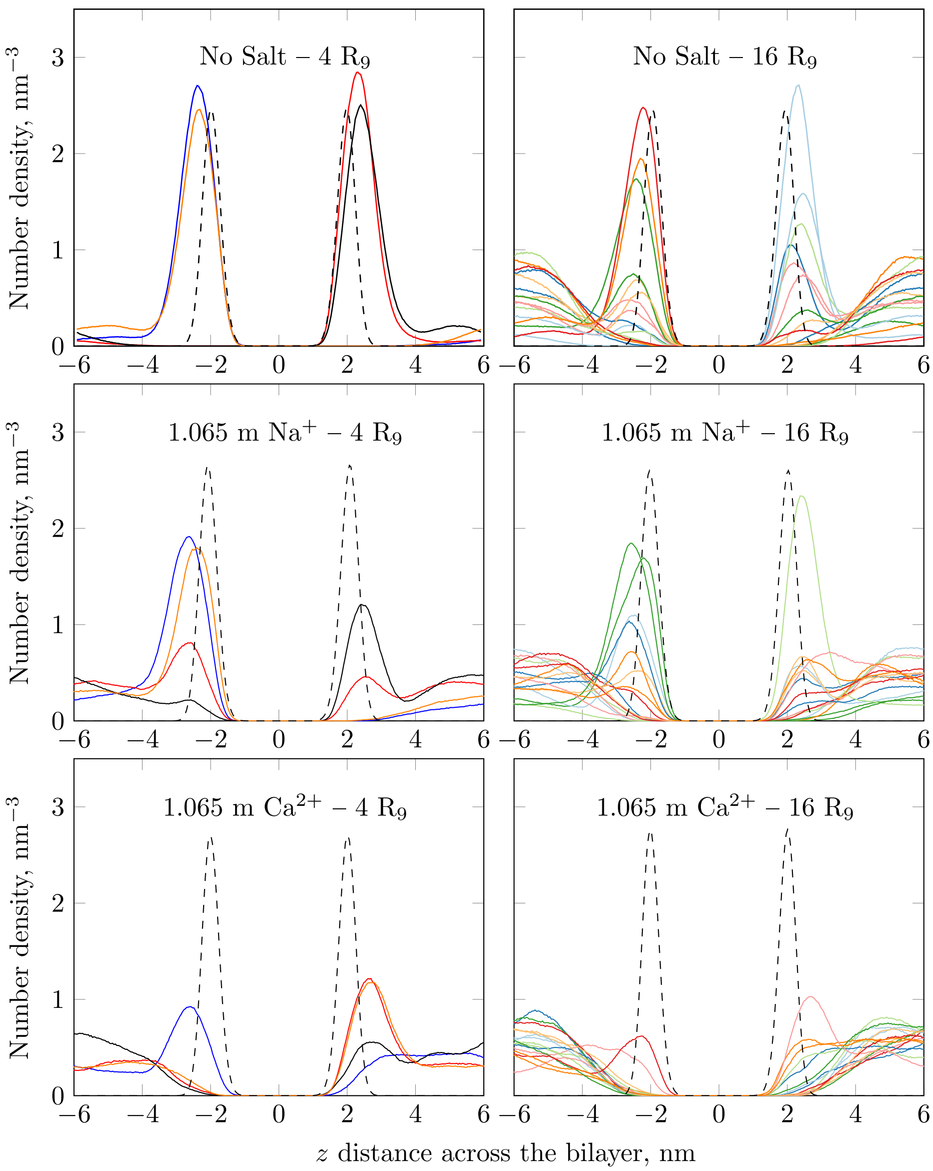

Figure S1: Asymmetric number density distribution of each R_9 peptide in the solution across the POPC bilayer. Each peptide is differently colored. Black dashed lines represent POPC phosphorus atom number density distribution.

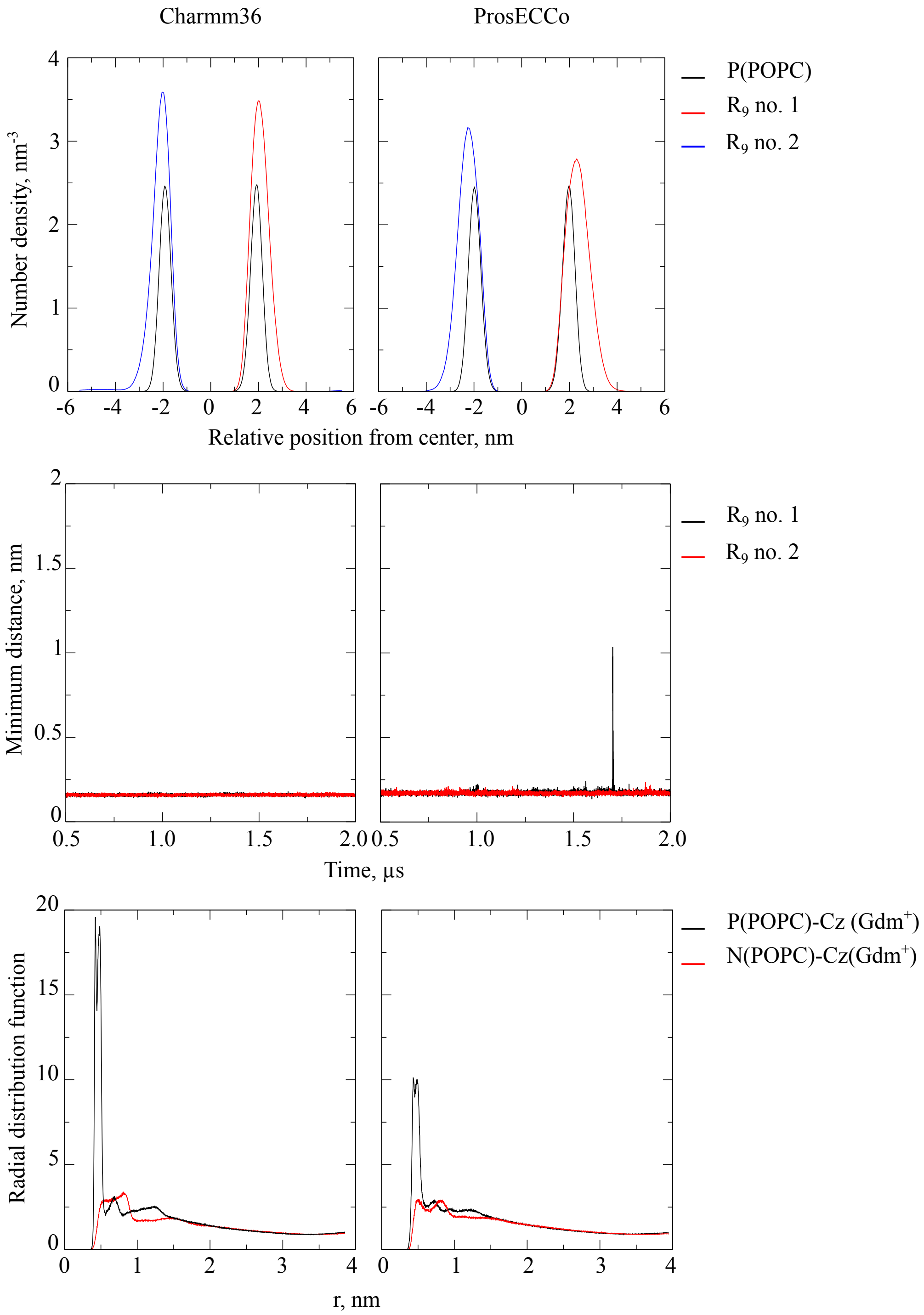

Figure S2: Molecular dynamics simulations for two R_9 peptides in salt-free solution with full charged vs. ProsECCo force field. Top panels: Number density distributions of each peptide across the POPC bilayer. Middle panels: Minimum distance of center of mass of single peptide to POPC lipid head groups. Bottom panels: Radial distributions functions between guanidinium cations of R_9 and P and N of POPC lipid, respectively.

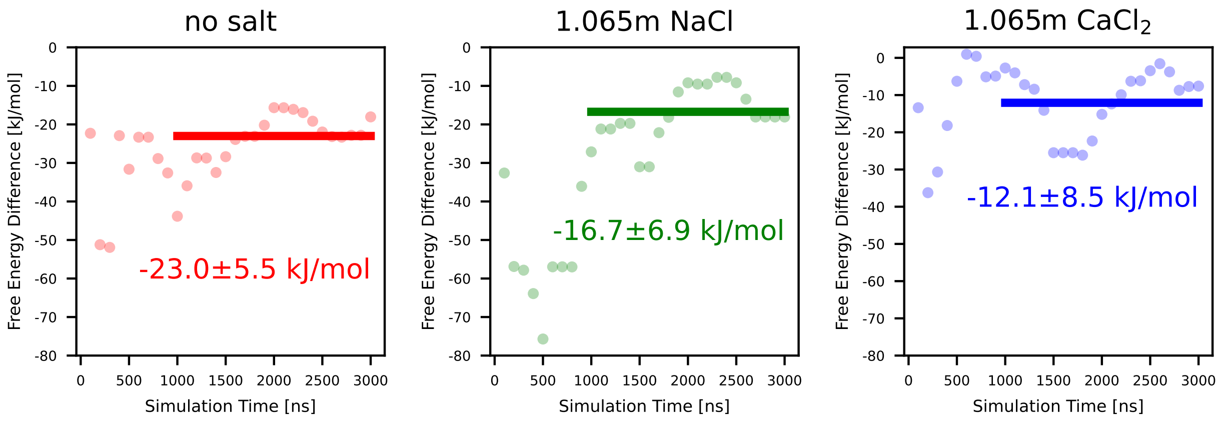

Figure S3: The time evolution of the adsorption free energy from well-tempered metadynamics simulations of R_9-containing systems. For each 100 ns window, the adsorption free energy was calculated as the difference between the lowest energy in the bound region of the two-dimensional energy profile (any energy point when each peptide was not further than 4 nm from the membrane) and the average energy of the non-bound region, i.e., both peptides are further than 4 nm from the surface. The solid lines are the averages of the adsorption free energies from the last two-thirds of simulations. The standard deviation is calculated from the block averaging over the five time-equal windows.

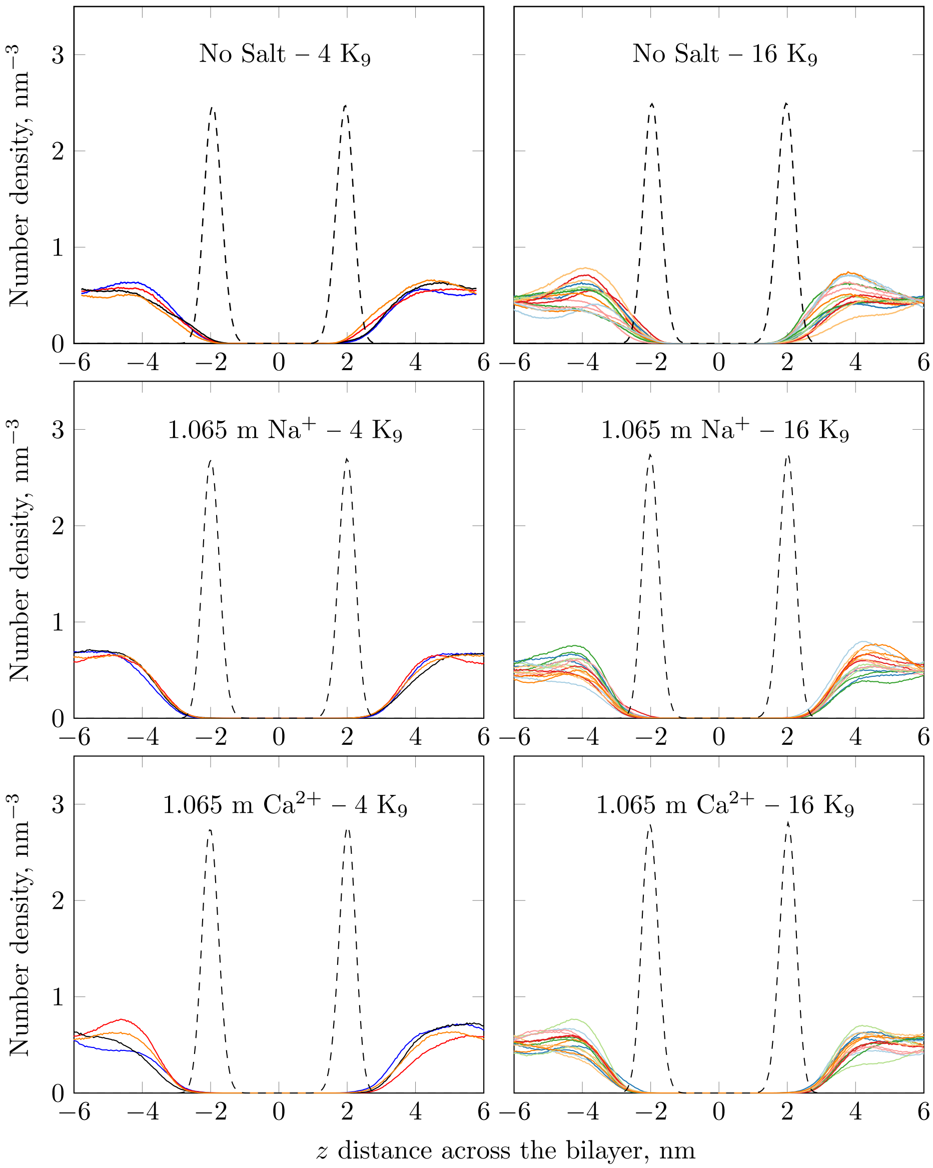

Figure S4: Asymmetric number density distribution of each K_9 peptide in the solution across the POPC bilayer. Each peptide is differently colored. Black dashed lines represent POPC phosphorus atom number density distribution.

Additionally, the convergence of all the unbiased MD simulations were examined to make sure that the obtained free energy values are extracted from equilibrium conditions where a suitable amount of binding and unbinding events take place. To do so, we calculated the minimum distances between each peptide and the nitrogen atoms of the choline head groups of POPC lipids during simulation time. The results are shown in Figure S5. For simplicity, we selected the unbiased simulation systems consisting of the lowest and the highest peptide concentrations (*i.e.* the number of peptides are 4 and 16 molecules, respectively) in the $NaCl/CaCl_2$ solutions at 0.0, 0.133, and 1.065 m as representatives.

As can be seen, in the majority of cases, each R_9 peptide exchanges between bound and unbound states. However, in the case of only 4 R_9 molecules, one of the peptides remains bound on the lipid surface throughout the 2 µs simulation time. This indicates that the extracted free energy estimates for these systems provide a lower bound for the free energy.

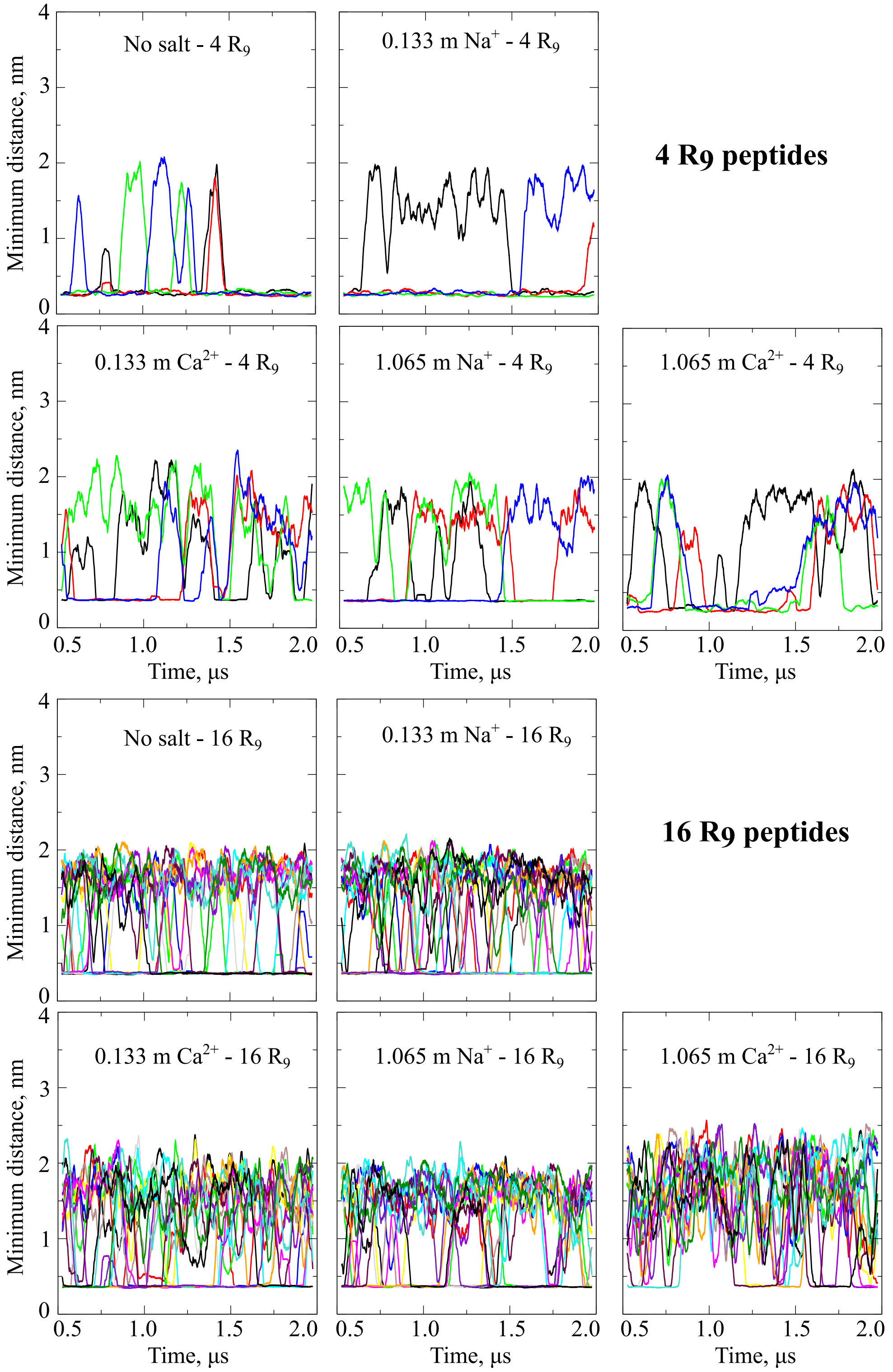

Figure S5: Calculated minimum distances between each R_9 peptide and the nitrogen atoms of the choline head groups of POPC during the simulation time. Each peptide is represented by a line of different color.

Table S1: List of systems are simulated in this study. The values shown in parentheses are corresponding molal concentrations.

Number of molecules			
Lipids	Salts	Peptides	Waters
200	0 (0.000)	2 (0.007)	15856
200	0 (0.000)	4 (0.014)	15856
200	0 (0.000)	6 (0.021)	15856
200	0 (0.000)	10 (0.035)	15856
200	0 (0.000)	13 (0.046)	15856
200	0 (0.000)	16 (0.056)	15856
200	19 (0.067)	2 (0.007)	15856
200	19 (0.067)	4 (0.014)	15856
200	19 (0.067)	6 (0.021)	15856
200	19 (0.067)	10 (0.035)	15856
200	19 (0.067)	13 (0.046)	15856
200	19 (0.067)	16 (0.056)	15856
200	38 (0.133)	2 (0.007)	15856
200	38 (0.133)	4 (0.014)	15856
200	38 (0.133)	6 (0.021)	15856
200	38 (0.133)	10 (0.035)	15856
200	38 (0.133)	13 (0.046)	15856
200	38 (0.133)	16 (0.056)	15856
200	76 (0.133)	2 (0.007)	15856
200	76 (0.266)	4 (0.014)	15856
200	76 (0.266)	6 (0.021)	15856
200	76 (0.266)	10 (0.035)	15856
200	76 (0.266)	13 (0.046)	15856
200	76 (0.266)	16 (0.056)	15856
200	152 (0.533)	2 (0.007)	15856
200	152 (0.533)	4 (0.014)	15856
200	152 (0.533)	6 (0.021)	15856
200	152 (0.533)	10 (0.035)	15856
200	152 (0.533)	13 (0.046)	15856
200	152 (0.533)	16 (0.056)	15856
200	304 (1.065)	2 (0.007)	15856
200	304 (1.065)	4 (0.014)	15856
200	304 (1.065)	6 (0.021)	15856
200	304 (1.065)	10 (0.035)	15856
200	304 (1.065)	13 (0.046)	15856
200	304 (1.065)	16 (0.056)	15856
